# Lymph node ratio is a superior predictor in surgically treated early-onset pancreatic cancer

**DOI:** 10.3389/fonc.2022.975846

**Published:** 2022-09-02

**Authors:** Yangyang Zheng, Zhenhua Lu, Xiaolei Shi, Tianhua Tan, Cheng Xing, Jingyong Xu, Hongyuan Cui, Jinghai Song

**Affiliations:** ^1^ Department of General Surgery, Department of Hepato-bilio-pancreatic Surgery, Beijing Hospital, National Center of Gerontology, Institute of Geriatric Medicine, Chinese Academy of Medical Sciences, Beijing, China; ^2^ Graduate School of Peking Union Medical College, Chinese Academy of Medical Sciences, Beijing, China

**Keywords:** early-onset pancreatic cancer, lymph node ratio, log odds of positive lymph nodes, examined lymph nodes, nomogram

## Abstract

**Background:**

The prognostic performance of four lymph node classifications, the 8th American Joint Committee on Cancer (AJCC) Tumor Node Metastasis (TNM) N stage, lymph node ratio (LNR), log odds of positive lymph nodes (LODDS), and examined lymph nodes (ELN) in early-onset pancreatic cancer (EOPC) remains unclear.

**Methods:**

The Surveillance, Epidemiology, and End Results (SEER) database was searched for patients with EOPC from 2004 to 2016. 1048 patients were randomly divided into training (n = 733) and validation sets (n = 315). The predictive abilities of the four lymph node staging systems were compared using the Akaike information criteria (AIC), receiver operating characteristic area under the curve (AUC), and C-index. Multivariate Cox analysis was performed to identify independent risk factors. A nomogram based on lymph node classification with the strongest predictive ability was established. The nomogram’s precision was verified by the C-index, calibration curves, and AUC. Kaplan–Meier analysis and log-rank tests were used to compare differences in survival at each stage of the nomogram.

**Results:**

Compared with the 8th N stage, LODDS, and ELN, LNR had the highest C-index and AUC and the lowest AIC. Multivariate analysis showed that N stage, LODDS, LNR were independent risk factors associated with cancer specific survival (CSS), but not ELN. In the training set, the AUC values for the 1-, 3-, and 5-year CSS of the nomogram were 0.663, 0.728, and 0.760, respectively and similar results were observed in the validation set. In addition, Kaplan–Meier survival analysis showed that the nomogram was also an important factor in the risk stratification of EOPC.

**Conclusion:**

We analyzed the predictive power of the four lymph node staging systems and found that LNR had the strongest predictive ability. Furthermore, the novel nomogram prognostic staging mode based on LNR was also an important factor in the risk stratification of EOPC.

## Introduction

Pancreatic cancer is a highly lethal solid organ malignancy with a poor 5-year overall survival (OS) rate approaching 10% and is the third leading cause of cancer-related death in America (1). With increased incidence and mortality rates, and difficulty in developing effective therapies for pancreatic cancer, this type of malignancy is expected to be the second-leading cause of cancer-related death in developed countries by 2040 (2). Although pancreatic cancer is often thought of as a disease of older adults, with a higher incidence in people aged 60 to 70 years, some retrospective studies have observed an increase in the incidence in younger age groups. Sung et al. observed a significant increase in the incidence of pancreatic cancer in people under 50 years of age (3). Tavakkoli et al. found that the incidence of pancreatic cancer increased by 44% in black and 57% in white patients aged 30-39 years, highlighting the emerging challenge of early-onset pancreatic cancer (EOPC) (4). Currently, there is no international standard definition of early onset pancreatic cancer. In recent years, early onset pancreatic cancer has been defined in literatures as pancreatic cancer diagnosed before the age of 40, 45, 50 years (5–8). Considering that researchers often use the criterion of age < 50 years for EOPC in literatures and the American Gastrointestinal Association Institute recommended that screening for pancreatic cancer in high-risk groups should begin at age 50, we have defined EOPC as pancreatic cancer diagnosed at an age of less than 50 years in this study (8–13). One study reported that EOPC can result in up to one-third of the total years lost to disease, suggesting its impact on overall life expectancy (14). Patients with EOPC often contribute to the extra global cancer burden by bringing about years of life lost because of premature death; thus, improving its prognosis may dramatically help reduce the global burden of disease.

Although the risk factors for EOPC are similar to those for average age-onset pancreatic cancer (AOPC), including obesity, smoking, and alcohol consumption, studies have further suggested that patients with EOPC may present unique clinical, pathological, and genomic features that may affect prognosis (8, 14–18). Some studies have also shown that patients with EOPC tend to be diagnosed at a later clinical stage. Consequently, these patients receive more aggressive treatment in the absence of significant comorbidities and have a better prognosis because of better functional reserve (8, 11, 19).

Among the predictors of postoperative survival, lymph node metastasis is a decisive factor for pancreatic cancer (20, 21). Therefore, accurate and efficient indicators for the evaluation of lymph node metastasis are necessary to provide individualized treatment and improve the prognosis of patients with EOPC. Little is known about the relationship between the prognosis of postoperative patients with EOPC and the new lymph node classifications. Nomogram is a predictive tool of visually assessing risk by providing a numerical estimate of the probability of a specific clinical event, while incorporating key factors of clinical outcome. Nomogram prognostic model has been used extensively in recent years for the prediction of most tumors. Some studies have shown that the predictive performance of nomogram is superior to that of TNM stage (22–24).

The purpose of the current study was to compare the predictive ability of four lymph node staging systems, namely, the 8th American Joint Committee on Cancer (AJCC) Tumor Node Metastasis (TNM) N stage, lymph node ratio (LNR), log odds of positive lymph nodes (LODDS), and examined lymph nodes (ELN), to screen the most effective factor in predicting cancer specific survival (CSS) in patients with EOPC who have undergone surgery and establish a novel nomogram prognostic staging model.

## Materials and methods

### Data collection in the SEER database

This retrospective study focused on patients diagnosed with EOPC who underwent radical surgery between 2004 and 2016. All the data involved in this study were retrieved from the Surveillance, Epidemiology, and End Results (SEER) 18 registry research database (SEER*Stat 8.3.9). SEER database is a federally funded, private information-free, publicly available cancer reporting system. We have been granted access to the database data (SEER Stat username: 14866-Nov2020).

Patients diagnosed with EOPC were identified according to the site codes (C25.0-25.9) and histologic codes (8010, 8020, 8021,8022, 8050, 8140, 8141, 8230, 8260, 8450, 8453, 8471,8480, 8481and 8500) of the International Classification of Diseases for Oncology, 3rd edition (ICD-O-3). This study defined early-onset disease as a diagnosis before the age of 50. The inclusion criteria were as follows (1) patients diagnosed with EOPC were identified according to the site codes (C25.0-25.9) and histologic codes (8010, 8020, 8021,8022, 8050, 8140, 8141, 8230, 8260, 8450, 8453, 8471,8480, 8481and 8500) of the International Classification of Disease for Oncology, 3rd edition (ICD-O-3); (2) patients diagnosed before the age of 50 years; (3) patients with complete lymph node biopsy records; (4) patients undergoing radical surgery; and (5) patients with a survival time of more than one month after surgery. The exclusion criteria were as follows: (1) an unconfirmed diagnosis by histopathology; (2) incomplete clinicopathological data; (3) patients who died of causes other than pancreatic cancer or an unknown cause; (4) pancreatic operation without lymph node harvest; and (5) incomplete or absent information about survival time, overall life status, or other characteristics.

Ultimately, 1,048 patients with EOPC were enrolled in this study. The patients were randomly divided into a training set (n = 733) and validation set (n = 315) at a ratio of 7:3 (**Table 1**). Data on the were obtained: sex, age, race, year of diagnosis, histological grade, SEER historic stage, histology, tumor size, AJCC TNM stage, T/N/M stage, ELN, positive lymph node (PLN), LNR, and LODDS. The calculation formulas for LNR and LODDS are as follows: LNR = PLN/ELN; LODDS = log [(PLN + 0.05)/(ELN-PLN + 0.05)]. TNM stages of patients with EOPC in the SEER database were updated to align with the 8th edition of the AJCC criteria. In this study, cancer specific survival (CSS), defined as the date of diagnosis to the date of death from pancreatic cancer, was set as the end event.

**Table 1:** Demographics and clinicopathologic characteristics of EOPC patients.

Variables	Total number, n (%)	Training cohort, n (%)	Validation cohort, n (%)	P-value
	(n = 1048)	(n = 733)	(n = 315)	
Sex				1
female	500 (47.7)	350 (47.7)	150 (47.6)	
male	548 (52.3)	383 (52.3)	165 (52.4)	
age				0.44
≤23	24 (2.3)	19 (2.6)	5 (1.6)	
24-49	1024 (97.7)	714 (97.4)	310 (98.4)	
race				0.21
White	799 (76.2)	564 (76.9)	235 (74.6)	
Black	138 (13.2)	88 (12.0)	50 (15.9)	
Other	111 (10.6)	81 (11.1)	30 (9.5)	
Tumor location				0.70
head	715 (68.2)	496 (67.7)	219 (69.5)	
body/tail	210 (20.0)	147 (20.1)	63 (20.0)	
other	123 (11.7)	90 (12.3)	33 (10.5)	
Grade				0.71
Well	169 (16.1)	119 (16.2)	50 (15.9)	
Moderate	516 (49.2)	368 (50.2)	148 (47.0)	
Poor	337 (32.2)	229 (31.2)	108 (34.3)	
Undifferentiated	26 (2.5)	17 (2.3)	9 (2.9)	
Chemotherapy				0.97
None/Unknown	277 (26.4)	193 (26.3)	84 (26.7)	
Yes	771 (73.6)	540 (73.7)	231 (73.3)	
Beam Radiation				0.65
None/Unknown	616 (58.8)	427 (58.3)	189 (60.0)	
Yes	432 (41.2)	306 (41.7)	126 (40.0)	
Type of pancreatectomy				0.66
PD	700 (66.8)	496 (67.7)	204 (64.8)	
DP	168 (16.0)	116 (15.8)	52 (16.5)	
TP	117 (11.2)	81 (11.1)	36 (11.4)	
others	63 (6.0)	40 (5.5)	23 (7.3)	
Tumor size (mm)				0.36
≤ 18	112 (10.7)	77 (10.5)	35 (11.1)	
>18	936 (89.3)	656 (89.5)	280 (88.9)	
8th AJCC T stage				0.29
T1	172 (16.4)	128 (17.5)	44 (14.0)	
T2	494 (47.1)	340 (46.4)	154 (48.9)	
T3	322 (30.7)	219 (29.9)	103 (32.7)	
T4	60 (5.7)	46 (6.3)	14 (4.4)	
8th AJCC N stage				0.22
N0	371 (35.4)	267 (36.4)	104 (33.0)	
N1	430 (41.0)	288 (39.3)	142 (45.1)	
N2	247 (23.6)	178 (24.3)	69 (21.9)	
8th AJCC M stage				1
M0	981 (93.6)	686 (93.6)	295 (93.7)	
M1	67 (6.4)	47 (6.4)	20 (6.3)	
8th AJCC TNM stage				0.72
I	210 (20.0)	149 (20.3)	61 (19.4)	
II	502 (47.9)	343 (46.8)	159 (50.5)	
III	269 (25.7)	194 (26.5)	75 (23.8)	
IV	67 (6.4)	47 (6.4)	20 (6.3)	
ELN				0.61
ELN1	172 (16.4)	117 (16.0)	55 (17.5)	
ELN2	876 (83.6)	616 (84.0)	260 (82.5)	
LNR				0.59
LNR1	397 (37.9)	284 (38.7)	113 (35.9)	
LNR2	300 (28.6)	210 (28.6)	90 (28.6)	
LNR3	351 (33.5)	239 (32.6)	112 (35.6)	
LODDS				0.40
LODDS1	403 (38.5)	286 (39.0)	117 (37.1)	
LODDS2	254 (24.2)	183 (25.0)	71 (22.5)	

EOPC, early-onset pancreatic cancer; PD, pancreaticoduodenectomy; DP, distal pancreatectomy; TP, total pancreatectomy; ELN, examined lymph nodes; LNR, positive lymph node ratio; LODDS, log odds of positive lymph nodes.

### Optimal cut-off points of the variables

The best cut-off values for age, tumor size, ELN, LNR, and LODDS were calculated using X-tile 3.6.1, based on the principles of maximum chi-squared value and minimum p-value (**Table S1**). The best cutoff point for age was 23 years, and the tumor size was 18 mm. As the best cut-off point for ELN was 6, ELN was divided into two groups: ELN1 (ELN ≤ 6) and ELN2 (ELN > 6). The best cut-off points for LNR were 0.04 and 0.17 mm, hence, LNR was divided into three groups, LNR1 (LNR ≤ 0.04), LNR2 (0.04 < LNR ≤ 0.17), and LNR3 (> 0.17). The best cut-off points for LODDS were -1.01 and -0.65 mm; thus, LODDS was divided into three groups: LODDS1 (LODDS ≤ -1.01), LODDS2 (−1.01 < LODDS ≤-0.65), and LODDS3 (LODDS > -0.65).

### Statistical analysis

Continuous variables were expressed as medians (quartiles), while ranked or categorical variables were presented as numbers (percentages). Kaplan–Meier curves and log-rank tests were used to assess the effectiveness of N stage, ELN, LNR, and LODDS for prognostic stratification of EOPC. The C-index, Akaike information criterion (AIC), and receiver operating characteristic (ROC) area under the ROC curve (AUC) were used to compare the predictive performance of N stage, ELN, LNR, and LODDS. Independent risk factors for EOPC were identified using univariate and multifactorial Cox analyses. Corresponding hazard ratio (HR) and 95% confidence intervals (CI) were also calculated. Based on the results of the multifactorial analysis, a nomogram of EOPC was constructed to predict 1-, 3-, and 5-year CSS. In addition, the predictive performance of the nomogram was validated using AUC, calibration curve, AIC, and C-index, and compared with the AJCC TNM staging system. Risk scores were calculated on the nomogram, and risk stratification was performed using X-tile version 3.6.1, with three stages (I, II, and III). Kaplan–Meier analysis and log-rank tests were used to compare differences in survival at each stage. The study was statistically analyzed using R software (version.4.0.3; The R Project for Statistical Computing, TX, USA; http://www.r-project.org). Statistically significant difference was set at two-tailed p < 0.05.

## Results

### Clinicopathological characteristics

From 2004 to 2016, 1,048 patients with radically resected EOPC were included in this study. They were randomly divided into a training set (n =733) and validation set (n = 315) at a ratio of 7:3. **Table 1** summarizes the clinical and pathological characteristics of the patients with EOPC. The median survival time was 20 months (IQR, 11–39 months), and the 1-, 3, and 5-year CSS rates were 74.6, 38.1, and 29.9%, respectively. Of the entire cohort (n =1048), 548 (52.3%) were men and 799 (76.2%) were white. A total of 1024 (97.7%) patients were aged > 23 years at the time of diagnosis, and the median age at diagnosis was 46 years (IQR, 42–48). A total of 712 (67.9%) patients had AJCC TNM stage I-II, and 67 (6.4%) patients had metastasis. There were 700 (66.8%) patients who underwent pancreatoduodenectomy (PD) and 117 (11.2%) who underwent total pancreatectomy (TP). At the same time, patients (677, 64.6%) had regional lymph node metastasis, and only 172 (16.4%) patients had an ELN ≤ 6.

### Comparison of four lymph node staging systems

**Figure 1:**
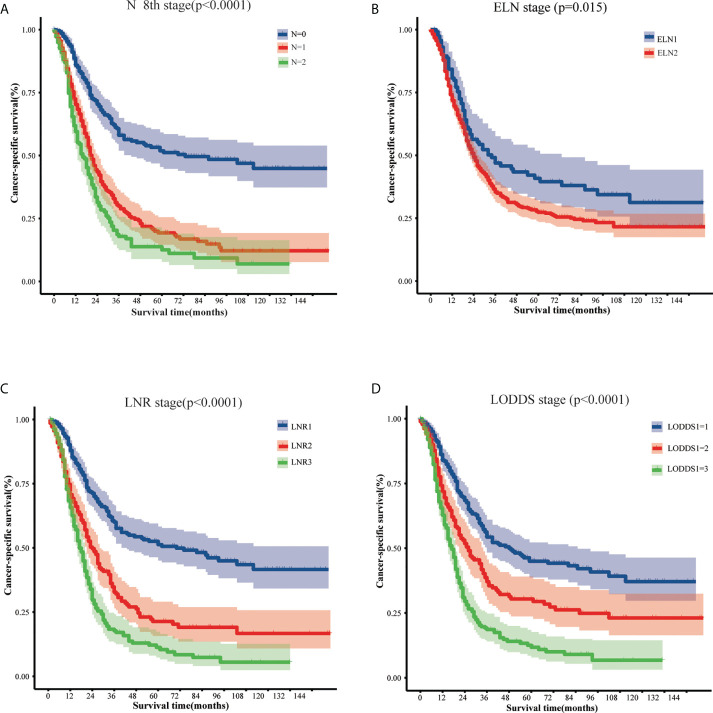
The cancer-specific survival of early-onset pancreatic cancer patients who underwent surgery stratified by different lymph node classifications: **(A)** 8th AJCC N stage, **(B)** the ELN stage, **(C)** the LNR stage, and **(D)** the LODDS stage. AJCC, American Joint Committee on Cancer; ELN, examined lymph nodes; LNR, lymph node ratio; LODDS, log odds of positive lymph nodes.

Univariate analysis based on the training set showed that 12 variables were strongly associated with cancer specific survival in patients with EOPC: sex, tumor location, grade, chemotherapy, tumor size, 8th AJCC T stage, 8th AJCC N stage, 8th AJCC M stage, 8th AJCC TNM stage, ELN, LNR, and LODDS (**Table 2**). In addition, Kaplan–Meier survival analysis and log-rank test revealed that the prognosis of patients with EOPC could be stratified by the 8th AJCC N stage, ELN, LNR, and LODDS (**Figure 1**).

**Table 2:** Univariate analysis of prognostic factors associated with cancer-specific survival for EOPC patients in the training cohort.

Variables	Univariate analysis	
	HR (95% CI)	P-value
Sex		
male	Reference	
female	0.679 (0.564-0.818)	**<0.001**
age		
≤23	Reference	
24-49	1.964 (0.931-4.145)	0.076
race		
White	Reference	
Black	0.948 (0.712-1.263)	0.717
Other	0.890 (0.653-1.213)	0.460
Tumor location		
head	Reference	
body/tail	0.599 (0.462-0.776)	**<0.001**
other	0.953 (0.716-1.268)	0.743
Grade		
Well	Reference	
Moderate	2.196 (1.605-3.007)	**<0.001**
Poor	3.050 (2.203-4.224)	**<0.001**
Undifferentiated	2.075 (1.048-4.107)	**0.036**
Chemotherapy		
None/Unknown	Reference	
Yes	1.759 (1.388-2.229)	**<0.001**
Beam Radiation		
None/Unknown	Reference	
Yes	1.157 (0.962-1.391)	0.121
Tumor size (mm)		
≤ 18	Reference	
>18	2.008 (1.428-2.823)	**<0.001**
8th AJCC T stage		
T1	Reference	
T2	1.630 (1.240-2.142)	**<0.001**
T3	1.403 (1.045-1.883)	**0.024**
T4	2.349 (1.539-3.587)	**<0.001**
8th AJCC N stage		
N0	Reference	
N1	2.488 (1.974-3.135)	**<0.001**
N2	3.350 (2.601-4.314)	**<0.001**
8th AJCC M stage		
M0	Reference	
M1	1.962 (1.389-2.773)	**<0.001**
AJCC TNM stage		
I	Reference	
II	1.909 (1.439-2.534)	**<0.001**
III	3.165 (2.346-4.270)	**<0.001**
IV	3.809 (2.512-5.777)	**<0.001**
ELN		
ELN1	Reference	
ELN2	1.378 (1.062-1.787)	**0.016**
LNR		
LNR1	Reference	
LNR2	2.204 (1.732-2.804)	**<0.001**
LNR3	3.328 (2.641-4.194)	**<0.001**
LODDS		
LODDS1	Reference	
LODDS2	1.661 (1.296-2.128)	**<0.001**
LODDS3	2.841 (2.279-3.542)	**<0.001**

HR, hazard ratio; CI, confidence interval.

Significant p-values in bold (P<0.05).

In the training set, the C-indices for the N stage, ELN, LODDS, and LNR were 0.618, 0.518, 0.614, and 0.623, respectively. The AIC values for N stage, ELN, LODDS, and LNR were 8138.614, 8248.666, 8145.589, and 8119.163, respectively. The AUC values of the N stage, ELN, LODDS, and LNR for 1-year CSS were 0.643, 0.532, 0.623, and 0.635, respectively. The AUC values for the 3- year CSS of N stage, ELN, LODDS, and LNR were 0.688, 0.535, 0.680, and 0.702, respectively. The AUC values of N stage, ELN, LODDS, and LNR for 5- year CSS were 0.712, 0.547, 0.688, and 0.731, respectively. In conclusion, LNR had the highest C-index and AUC, with the lowest AIC, indicating that LNR had better predictive performance than N stage, ELN, and LODDS for CSS in patients with EOPC (**Table 3**). Based on the results of the univariate analysis combined with the same confounders, the N stage, ELN, LODDS, and LNR were incorporated into four different Cox regression models separately. Multivariate Cox regression analysis suggested that grade, tumor size, AJCC 8th M stage, AJCC 8th N stage, LODDS, LNR were independent prognostic indicators for CSS (**Table S2**; p < 0.05). However, ELN were not significantly associated with the prognosis of EOPC (**Table S2**; p = 0.30).

**Table 3:** Prognostic efficiency of different lymph node staging systems.

Systems	C-index	AIC	AUC		
			1-year CSS	3-year CSS	5-year CSS
N stage	0.618	8138.614	0.643	0.688	0.712
ELN	0.518	8248.666	0.532	0.535	0.547
LODDS	0.614	8145.589	0.623	0.680	0.688
LNR	**0.623**	8119.163	0.635	0.702	0.731

C-index, concordance index; AIC, Akaike information criterion; AUC, area under the receiver operating characteristic curve.

Significant values in bold.

### Construction and validation of the prognostic nomogram for CSS

**Figure 2:**
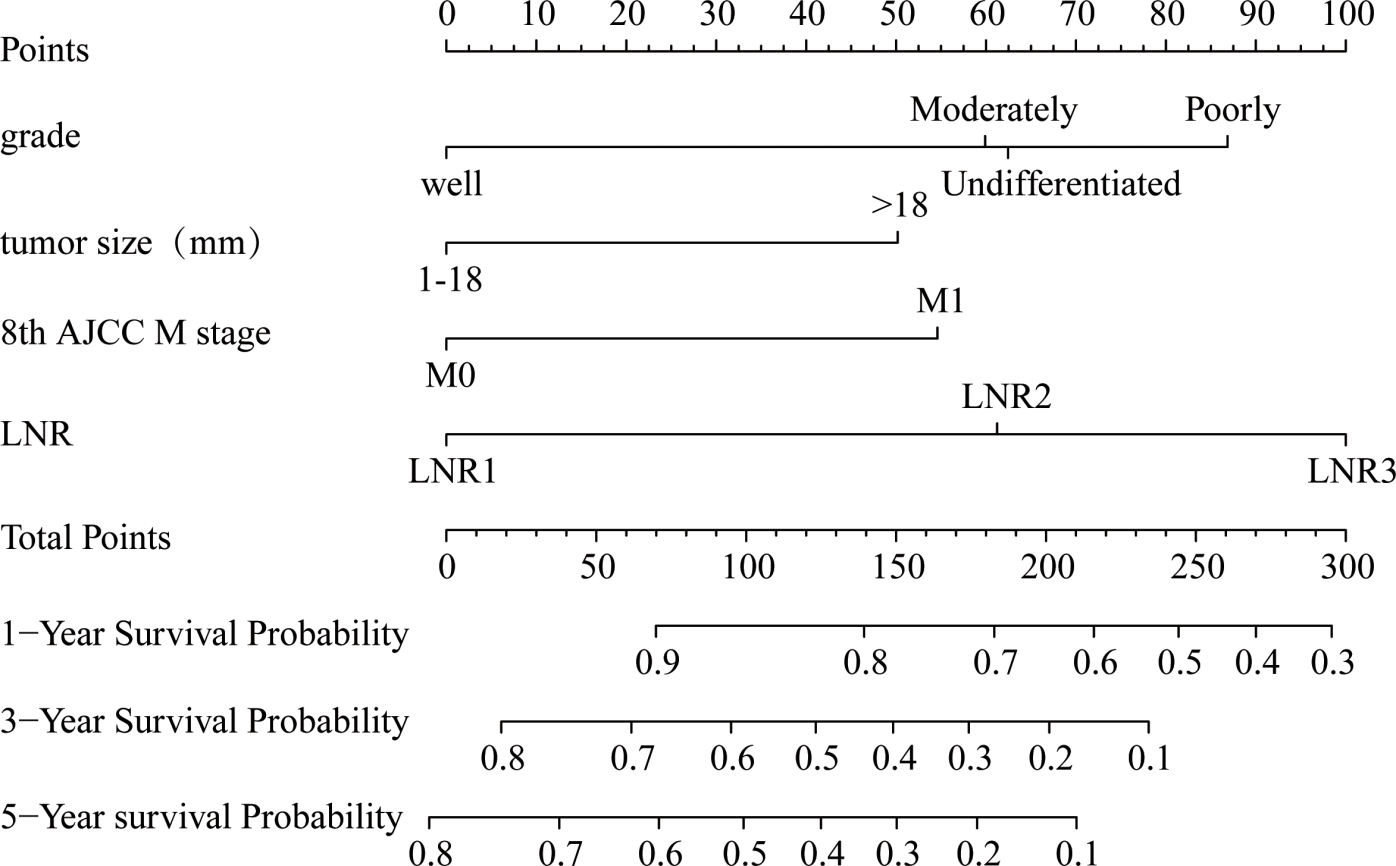
Nomogram for predicting the 1-, 3- and 5-year probabilities of cancer-specific survival in surgically treated early-onset pancreatic cancer patients in the training cohort. The nomogram was constructed by grade, tumor size, 8th AJCC M stage, and LNR. LNR, lymph node ratio; AJCC, American Joint Committee on Cancer.

A nomogram model for predicting cancer specific survival in patients with EOPC was developed (**Figure 2**) based on the above four independent risk factors: grade, tumor size, 8th AJCC M stage, and LNR. As shown in **Figure 2**, each factor of these variables was given a score on the point of the scale. Grades of differentiation well, moderate, poor, and undifferentiated were scored as 0, 60, 87 and 62, respectively. A tumor size ≤ 18 mm was scored as 0 and a tumor size > 18 mm was scored as 50. 8th AJCC M0 was scored as 0, and 8th AJCC M1 was scored as 55. LNR1 (LNR ≤ 0.04), LNR2 (0.04 < LNR ≤ 0.17), and LNR3 (> 0.17) were scored as 0, 61,100, respectively. By summing the scores for each variable, we can predict the CSS rates of 1-, 3-, and 5-year of patients with EOPC. For example, the probabilities of CSS rates of 1-, 3-, and 5-year for EOPC patients with Well-differentiated, tumor size ≤ 18 mm, LNR1 (LNR ≤ 0.04) and M0 ranged from 0.9 to 1, 0.8 to 1 and 0.8 to 1, respectively. However, the probabilities of CSS rates of 1-, 3-, and 5-year for EOPC patients with undifferentiated, tumor size > 18 mm, LNR3 (LNR > 0.17) and M1 ranged from 0.4 to 0.5, 0 to 0.1, and 0 to 0.1, respectively.

**Figure 3:**
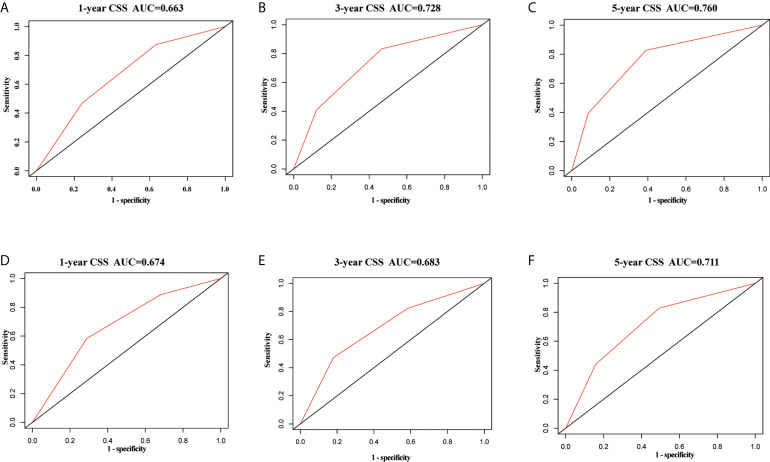
ROC curves of the nomogram for predicting cancer-specific survival in surgically treated early-onset pancreatic cancer patients. ROC curves of the nomogram predicting CSS for 1-year **(A)**, 3-year **(B)**, and 5-year **(C)** in the training set; ROC curves of the nomogram predicting CSS for 1-year **(D)**, 3-year **(E)**, and 5-year **(F)** in the validation set. ROC, receiver operating characteristic; CSS, cancer-specific survival.

**Figure 4:**
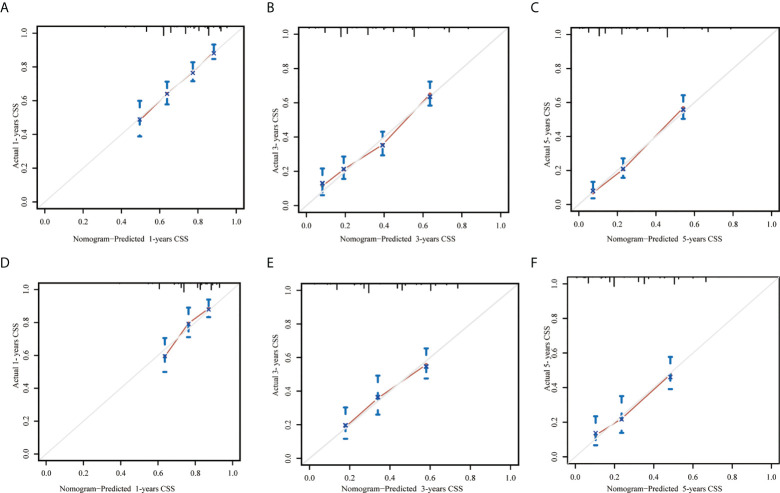
Calibration curves for predicting 1-, 3- and 5-year cancer-specific survival in patients with early-onset pancreatic cancer after surgery in the training set **(A–C**, respectively**)** and in the internal validation set **(D–F**, respectively**)**. The X-axis represented the nomogram-predicted probability of CSS and the Y-axis represented actual observed survival. The diagonal grey line represents an ideal evaluation where the predicted probabilities were identical to that of actual observed. CSS, cancer-specific survival.

In the training set, the AUC values of the nomogram were 0.663, 0.728, and 0.760, respectively (**Figures 3A–C**), for predicting 1-, 3-, and 5- year CSS in patients with EOPC. Similar outcomes were observed in the validation set, where the AUC values of the nomogram were 0.674, 0.683, and 0.711 for 1-, 3-, and 5-year CSS, respectively (**Figures 3D–F**). An optimal agreement was demonstrated by the calibration curves between the nomogram-predicted and measured 1-, 3-, and 5-year CSS in both the training (**Figures 4A–C**) and validation sets (**Figures 4D–F**).

To compare the performances of the nomogram and AJCC TNM stage, the C-index and AIC were calculated for both the training and validation cohorts (**Table 4**). In the training set, the C-indices of the nomogram and AJCC TNM stage for CSS prediction were 0.674 and 0.615, respectively. The AIC of the nomogram and AJCC TNM stage for CSS prediction were 5284 and 5370 respectively. The C-index of the nomogram was higher, and its AIC was lower than that of the AJCC TNM staging system. The results in the validation cohort were similar. The C-index of the nomogram was 0.668, which was higher than the AJCC TNM 0.594, and the AIC of the nomogram was 1955, which was lower than that of AJCC TNM 1997. In summary, these indicators demonstrated that the nomogram prognostic model showed superior performance to the AJCC TNM staging system.

**Table 4:** Prognostic efficiency of nomogram prognostic model and the 8th TNM.

Systems	C-index		AIC	
	Training cohort(n = 733)	Validation cohort (n = 315)	Training cohort (n = 733)	Validation cohort(n = 315)
Nomogram	**0.674**	**0.668**	**5284**	**1955**
8th TNM	0.615	0.594	5370	1997

C-index, concordance index; AIC, Akaike information criterion.

Significant values in bold.

### Risk stratification based on the nomogram

**Figure 5:**
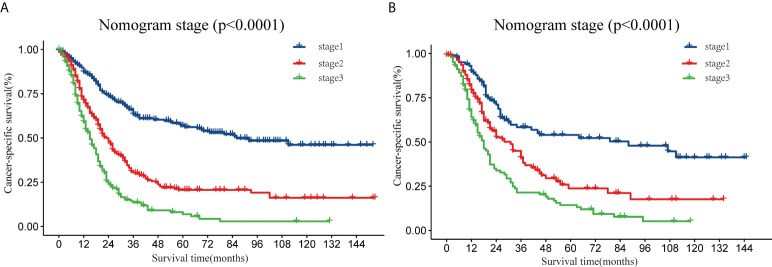
Analysis of the prognostic significance of the nomogram in surgically treated early-onset pancreatic cancer patients. Kaplan–Meier curves of CSS for all patients stratified by risk scores predicted by the nomograms in training set **(A)** and in the validation set **(B)**. CSS, cancer-specific survival.

In addition, the X-Tile software was used to determine the optimal cut-off values and establish a risk grading system. All patients were classified as low-risk (stage I, score: 0–136), medium-risk (stage II, score: 137–203), and high-risk (stage III, score: 204–292). Theoretically, the total scores range from 0 to 292. Kaplan–Meier curves (**Figure 5**) showed that the risk classification system had good stratification and differentiation in the training and validation sets, respectively.

## Discussion

Recent studies have shown that lymph node metastasis affects the surgical-pathological staging, treatment planning, and prognosis of pancreatic cancer, making it important to properly assess the status of lymph node metastasis (25, 26). To improve the accuracy of predicting pancreatic cancer prognosis, the American Joint Committee on Cancer (AJCC) published the 8th edition of the TNM classification. The 8th edition for the first time divided pancreatic cancer patients with lymph node metastases into three groups: N0 with no regional lymph nodes with metastases, N1 with one to three regional lymph nodes with metastases, and N2 with more than or equal to four regional lymph nodes with metastases. This reclassification emphasizes the importance of the number of positive lymph nodes in pancreatic cancer staging. Currently, the 8th AJCC TNM N stage remains the gold standard for assessing the status of lymph node metastasis for postoperative staging of pancreatic cancer. However, N staging is highly dependent on the number of positive lymph nodes, which is directly influenced by the total number of examined lymph nodes (ELN). Valsangkar et al. showed that the prognostic accuracy of any lymph node variable depends on the total number of examined lymph nodes (27). To ensure the accuracy of the N stage, the 8th AJCC guidelines state that the minimum number of lymph nodes to be detected should not be less than 12. However, in clinical practice, the number of retrieved lymph nodes depends on several factors, such as the extent of lymph node clearance, the operator’s skill and experience, the condition of the specimen, and individual patient variation. Moreover, among patients following neoadjuvant chemotherapy, destruction and fibrosis of lymphatic vessels and lymph nodes are observed, which increases the rate of negative pathological lymph nodes and makes lymph node detection more difficult. For example, in this study, 37.4% of patients did not meet the criteria for a minimum of 12 lymph nodes to be detected, which could interfere with the performance of the 8th N stage.

Published retrospective studies have observed that a higher ELN is often associated with better prognosis in patients with pancreatic cancer, particularly in N0 patients. Zhu et al. analyzed 10,910 patients from the National Cancer Database and found that a higher ELN correlated with better survival, and the survival benefits of adjuvant radiation therapy may not compensate for inadequate lymph node dissection (28). A study conducted by Malleo et al. suggested that, among lymph node-negative patients, the 5-year survival rate was significantly lower in patients with ELN < 20 than in those with ELN ≥ 20 (33.5% vs. 67.6%) (29). ELN was recognized as an important prognostic factor for two reasons. First, a higher number of ELN increases the chance of eradicating isolated tumor cells and micrometastases in the lymph nodes (30–32). Second, correct regional lymph node staging can only be obtained based on adequate ELN (28, 29, 33, 34). To date, there is no consensus on the recommendations for minimum ELN numbers in pancreatic cancer patients. Several retrospective analyses have attempted to establish a standard, with recommendations for minimum ELN varying widely from 11 to 17 (33, 35–37).

As young people are often not considered to be at high risk for pancreatic cancer, early-onset pancreatic cancer can easily be overlooked, and the diagnosis is delayed. Ordonez et al. reported that early-onset pancreatic cancer tended to present with a later stage (stage 3 or 4 cancer) of disease than AOPC (62.1% vs. 55.2%; p < 0.001) (8). Previous studies have shown that patients with EOPC tend to receive more aggressive treatment in the absence of significant comorbidities and have better functional reserve. Saadat et al. found that EOPC patients received more chemotherapy (38% vs. 29%), chemoradiation (12% vs. 9.2%), and multimodal treatment (21% vs. 15%) compared with AOPC (11). Receiving more chemotherapy preoperatively tends to increase the difficulty of lymph node detection. This may explain the cut-off value of 6 for the ELN in this study, which is much smaller than the ELN in other studies. In this study, ELN was compared with other staging systems, and the predictive value of the model was assessed.

In recent years, LNR and LODDS staging systems have been gradually proposed in academia. As LNR and LODDS take into account both the number of positive lymph nodes and the number of examined lymph nodes, they are considered to have better staging ability for metastatic lymph nodes. Wu et al. retrospectively analyzed 177 patients who underwent pancreaticoduodenectomy and found that the LNR was an independent prognostic factor for pancreatic cancer (38). Slidell et al. divided 4,005 patients into LNR = 0, 0 < LNR ≤ 0.2, 0.2 < LNR ≤ 0.4, and LNR > 0.4, for survival analysis, and found that there was a significant difference in the overall survival among the four groups (39).The LNR combines the prognostic impact of both the number of positive lymph nodes and ELN, and reduces stage migration to some extent; however, it loses its inherent ability when N is 0 or when all intraoperative lymph nodes are detected positively, with an LNR value of 0 or 1. Some scientists have attempted to assess lymph node status using a new metric, which is the log odds of positive lymph nodes (LODDS).La et al. showed that LODDS and LNR were independent prognostic factors in patients with pancreatic cancer and that LODDS had a better prognostic power than LNR in N0 patients (40). However, after summarizing comparative studies of LNR and LODDS, we found no consensus on the advantages of LNR and LODDS. Ramacciato et al. found that ELN, LNR, and LODDS were useful for further prognostic stratification of N1 patients undergoing pancreatic resection combined with portal/superior mesenteric vein resection, while no one method was found to be superior to the other (41). Lee et al. evaluated several current lymph node-related prediction models in 2,584 patients and concluded that LNR and AJCC 8th edition N staging had better predictive value than LODDS regardless of the number of lymph nodes detected, whether R1 resection was performed, and the extent of surgical resection (42). Zou et al. found no significant difference in the concordance index (C-index) of the two prediction models by comparing LNR with N-staging, but N-staging was found to be clinically more accessible than LNR (43).

Recent research has shown that cancer is trending towards the younger population (44). As young people are not already a high-risk group for cancer, it is easy to delay diagnosis and treatment among young cancer patients. Constructing a postoperative predictive model in the hope of helping clinicians identify high-risk patients early and intervene as soon as possible is critical for improving the prognosis of patients with EOPC. However, no study has examined the prognostic significance and staging accuracy of the four current lymph node staging systems, 8th AJCC T stage,8th AJCC N stage, lymph node ratio (LNR), log odds of positive lymph nodes (LODDS), and examined lymph nodes (ELN) in early-onset pancreatic cancer.

In this study, univariate analysis showed that sex, tumor location, grade, chemotherapy, tumor size, 8th AJCC N stage, 8th AJCC M stage, AJCC TNM stage, ELN, LNR, and LODDS were strongly associated with CSS in patients with EOPC. The prognostic predictive performance of the LNR stage was also found to be superior to that of the 8th AJCC TNM N stage, ELN, and LODDS, according to an evaluation criterion consisting of the C-index, AIC, and AUC values. Through multivariate Cox regression analyses, grade, tumor size, AJCC 8th M stage, AJCC 8th N stage, LODDS, LNR were verified as independent prognostic predictors for CSS with early-onset pancreatic cancer (p < 0.05). However, ELN were not significantly associated with the prognosis of EOPC (p = 0.30).

Based on four independent risk factors, including grade, tumor size, 8th AJCC M stage, and LNR, we constructed and validated a novel nomogram. A new LNR stage was obtained by stratifying the LNR risk score according to the principles of maximum chi-squared and minimum p-values using the X-tile software. Furthermore, Kaplan–Meier survival analysis showed that the LNR stage was not only strongly associated with the prognosis of EOPC but was also an important factor in the risk stratification of early-onset pancreatic cancer. The higher the LNR stage, the lower the CSS. Dai et al. confirmed similarly that a higher LNR was an independent prognostic factor of CSS based on their retrospective study of 1,386 pancreatic cancer (<45 years) (6). LNR has been also regarded as an independent prognostic factor that is strongly associated with the prognosis of various malignancies such as oral cancer, esophageal cancer, breast cancer and medullary thyroid cancer, etc (45–48). In studies of esophageal cancer and medullary thyroid cancer, LNR showed superior prognostic performance than N stage (46, 48).

To our knowledge, this study demonstrated for the first time that LNR is a more reliable prognostic staging indicator than AJCC 8th edition N-stage, ELN, and LODDS in early-onset pancreatic cancer. In addition, the performance of the nomogram was demonstrated to be more accurate and intuitive than that of the 8th AJCC TNM staging system. However, this study has several limitations. First, despite the large sample size, this was a retrospective study, and the findings need to be validated by prospective studies. Second, because this study used the SEER database, selection bias was likely to exist in our study. Third, the type of surgical resection (R0, R1, or R2) and other clinical information affecting patient survival (e.g., jaundice, CA199, neoadjuvant/adjuvant regimens) were not available from the SEER database; hence, they could not be included in the Cox regression model. Fourth, recurrence information was not available for SEER, and disease-free survival could not be assessed in this study.

## Conclusions

In conclusion, this study retrospectively analyzed 1,048 patients with early-onset pancreatic cancer from the SEER database. Our results demonstrated that the LNR stage yielded superior prognostic efficiency compared with the 8th AJCC TNM N stage, LODDS, and ELN. In addition, N stage, LODDS, LNR were independent risk factors associated with cancer specific survival (CSS), but not ELN. The nomogram prognostic model based on LNR for predicting cancer specific survival in EOPC can be used to stratify patients with EOPC at high and low risk. This may complement the current 8th AJCC TNM staging system and help clinicians provide patients with EOPC with more individualized follow-up and treatment. Well-designed prospective clinical trials with appropriate sample sizes are required to verify these conclusions.

## Data availability statement

The raw data supporting the conclusions of this article will be made available by the authors, without undue reservation.

## Author contributions

YZ were involved in the final data collection, analysis, and manuscript writing. ZL, XS, and TT were involved in the data collection, ZL, CX, JX, and HC were involved in the data analysis. JS was involved in study design and responsible for the entire research project. All authors have agreed on the journal to which the article has been submitted, agree to be accountable for all aspects of the work criteria, made a significant contribution to the work reported, contributed to the article, and approved the submitted version.

## Acknowledgments

The authors give special thanks to the National Cancer Institute for sharing the SEER data.

## Conflict of interest

The authors declare that the research was conducted in the absence of any commercial or financial relationships that could be construed as a potential conflict of interest.

## Publisher’s note

All claims expressed in this article are solely those of the authors and do not necessarily represent those of their affiliated organizations, or those of the publisher, the editors and the reviewers. Any product that may be evaluated in this article, or claim that may be made by its manufacturer, is not guaranteed or endorsed by the publisher.

## Abbreviations

AIC, Akaike information criterion; AJCC, American Joint Committee on Cancer; AOPC, Average age-onset pancreatic cancer; AUC, Area under the curve; CI, Confidence interval; CSS, Cancer specific survival; ELN, Examined lymph nodes; HR, Hazard ratio; LODDS, Log odds of positive lymph nodes; LNR, Lymph node ratio; PLN, Positive lymph node; ROC, Receiver operating characteristic; SEER, Surveillance Epidemiology and End Results; TNM, Tumor Node Metastasis (TNM).
