# Supplementary material for: Lymph node ratio is a superior predictor in surgically treated early-onset pancreatic cancer
Source: Front Oncol. 2022 Sep 2;12:975846. doi: 10.3389/fonc.2022.975846 (PMC9479329; doi:10.3389/fonc.2022.975846)
Supplement: Supplementary file 1 [file DataSheet_1.docx]

**Table S1 X-tile analysis of** **optimal cut-off values for continuous variables.**

| Variables | cut-off values | groups |
| --- | --- | --- |
| Age (years) | 23 | ≤23 |
|  |  | 24-49 |
| tumor size (mm) | 18 | ≤ 18 |
|  |  | >18 |
| ELN | 6 | ELN1 (ELN ≤ 6) |
|  |  | ELN2 (ELN > 6) |
| LNR | 0.04 | LNR1 (LNR ≤ 0.04) |
|  | 0.17 | LNR2 (0.04 < LNR ≤ 0.17) |
|  |  | LNR3 (> 0.17) |
| LODDS | -1.01 | LODDS1 (LODDS ≤ -1.01) |
|  | -0.65 | LODDS2 (−1.01 < LODDS ≤-0.65) |
|  |  | LODDS3 (LODDS > -0.65) |

*ELN, examined lymph nodes; LNR, positive lymph node ratio; LODDS, log odds of positive lymph nodes.*

**Table S2 Multivariate Cox regression analysis of prognostic factors associated with cancer-specific survival for EOPC patients in the training cohort. Significant p-values are in bold (P<0.05).**

| Variables | N stage |  |  | ELN |  |  | LNR |  |  | LODDS |  |
| --- | --- | --- | --- | --- | --- | --- | --- | --- | --- | --- | --- |
|  | HR (95%CI) | P-value |  | HR (95%CI) | P-value |  | HR (95%CI) | P-value |  | HR (95%CI) | P-value |
| Sex |  |  |  |  |  |  |  |  |  |  |  |
| male | Reference |  |  | Reference |  |  | Reference |  |  | Reference |  |
| female | 0.872 (0.721-1.056) | 0.161 |  | 0.775 (0.642-0.936) | **0.008** |  | 0.866(0.716-1.059) | 0.141 |  | 0.841 (0.695-1.018) | 0.075 |
| Tumor location |  |  |  |  |  |  |  |  |  |  |  |
| head | Reference |  |  | Reference |  |  | Reference |  |  | Reference |  |
| body/tail | 0.859 (0.651-1.133) | 0.282 |  | 0.729 (0.552-0.962) | **0.025** |  | 0.877(0.664-1.159) | 0.356 |  | 0.783 (0.594-1.032) | 0.083 |
| other | 1.095 (0.811-1.479) | 0.552 |  | 1.056 (0.781-1.428) | 0.724 |  | 1.142(0.846-1.543) | 0.386 |  | 1.138 (0.843-1.538) | 0.398 |
| Grade |  |  |  |  |  |  |  |  |  |  |  |
| Well | Reference |  |  | Reference |  |  | Reference |  |  | Reference |  |
| Moderate | 1.897 (1.362-2.641) | **<0.001** |  | 1.817 (1.308-2.525) | **<0.001** |  | 1.856(1.332-2.587) | **<0.001** |  | 1.814 (1.303-2.527) | **<0.001** |
| Poor | 2.415 (1.708-3.414) | **<0.001** |  | 2.445 (1.731-3.454) | **<0.001** |  | 2.490(1.757-3.5280 | **<0.001** |  | 2.586 (1.826-3.663) | **<0.001** |
| Undifferentiated | 1.952 (0.971-3.926) | 0.060 |  | 1.853 (0.925-3.714) | 0.082 |  | 1.934(0.961-3.892) | 0.064 |  | 1.889 (0.940-3.799) | 0.074 |
| Chemotherapy |  |  |  |  |  |  |  |  |  |  |  |
| None/Unknown | Reference |  |  | Reference |  |  | Reference |  |  | Reference |  |
| Yes | 0.995 (0.764-1.298) | 0.973 |  | 1.235 (0.952-1.602) | 0.113 |  | 1.042(0.081-1.357) | 0.757 |  | 1.125 (0.867-1.459) | 0.375 |
| Tumor size (mm) |  |  |  |  |  |  |  |  |  |  |  |
| ≤ 18 | Reference |  |  | Reference |  |  | Reference |  |  | Reference |  |
| >18 | 1.815 (1.141-2.887) | **0.012** |  | 1.927 (1.207-3.078) | **0.006** |  | 1.837(1.522-2.928) | **0.011** |  | 1.863 (1.167-2.975) | **0.009** |
| 8th AJCC T stage |  |  |  |  |  |  |  |  |  |  |  |
| T1 | Reference |  |  | Reference |  |  | Reference |  |  | Reference |  |
| T2 | 0.933 (0.649-1.343) | 0.710 |  | 0.986 (0.683-1.423) | 0.939 |  | 0.904(0.627-1.304) | 0.589 |  | 0.903 (0.625-1.304) | 0.586 |
| T3 | 0.967 (0.660-1.418) | 0.864 |  | 0.967 (0.658-1.422) | 0.865 |  | 0.922(0.628-1.354) | 0.678 |  | 0.911 (0.619-1.341) | 0.638 |
| T4 | 1.470 (0.911-2.371) | 0.115 |  | 1.419 (0.875-2.300) | 0.156 |  | 1.381(0.853-2.236) | 0.189 |  | 1.398 (0.861-2.268) | 0.175 |
| 8th AJCC M stage |  |  |  |  |  |  |  |  |  |  |  |
| M0 | Reference |  |  | Reference |  |  | Reference |  |  | Reference |  |
| M1 | 1.983 (1.391-2.828) | **<0.001** |  | 1.993 (1.398-2.842) | **<0.001** |  | 1.740(1.213-2.93) | **0.003** |  | 1.634 (1.142-2.339) | **0.007** |
| 8th AJCC N stage |  |  |  |  |  |  |  |  |  |  |  |
| N0 | Reference |  |  |  |  |  |  |  |  |  |  |
| N1 | 2.152 (1.678-2.758) | **<0.001** |  |  |  |  |  |  |  |  |  |
| N2 | 2.833(2.162-3.713) | **<0.001** |  |  |  |  |  |  |  |  |  |
| ELN |  |  |  |  |  |  |  |  |  |  |  |
| ELN1 |  |  |  | Reference |  |  |  |  |  |  |  |
| ELN2 |  |  |  | 1.151 (0.882-1.503) | 0.300 |  |  |  |  |  |  |
| LNR |  |  |  |  |  |  |  |  |  |  |  |
| LNR1 |  |  |  |  |  |  | Reference |  |  |  |  |
| LNR2 |  |  |  |  |  |  | 1.826(1.411-2.362) | **<0.001** |  |  |  |
| LNR3 |  |  |  |  |  |  | 2.808(2.192-3.596) | **<0.001** |  |  |  |
| LODDS |  |  |  |  |  |  |  |  |  |  |  |
| LODDS1 |  |  |  |  |  |  |  |  |  | Reference |  |
| LODDS2 |  |  |  |  |  |  |  |  |  | 1.480 (1.149-1.906) | **0.002** |
| LODDS3 |  |  |  |  |  |  |  |  |  | 2.470 (1.965-3.105) | **<0.001** |

*EOPC, early-onset pancreatic cancer;* *PD: pancreaticoduodenectomy; DP: distal pancreatectomy; TP: total pancreatectomy; ELN, examined lymph nodes; LNR, positive lymph node ratio; LODDS, log odds of positive lymph nodes; HR, hazard ratio; CI, confidence interval.*
